# Stathmin drives virus-induced metastasis

**DOI:** 10.18632/oncotarget.5961

**Published:** 2015-10-04

**Authors:** Adrian Whitehouse, Andrew Macdonald

**Affiliations:** School of Molecular and Cellular Biology, Faculty of Biological Sciences and Astbury Centre for Structural Molecular Biology, University of Leeds, Leeds, LS2 9JT, United Kingdom

**Keywords:** virus, metastasis, stathmin

**Figure 1:**
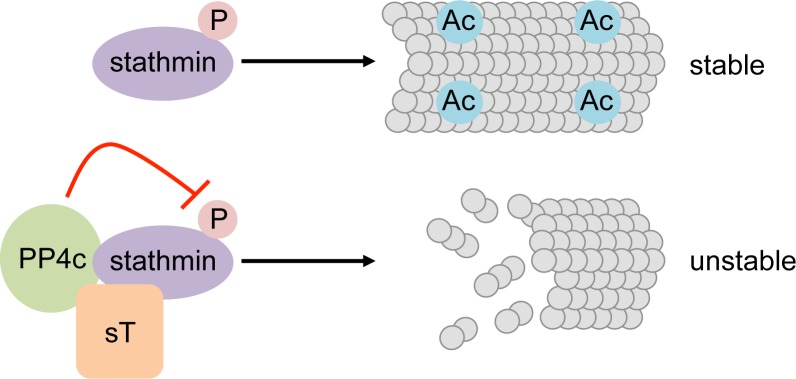
Stathmin is a phosphorylation regulated tubulin binding protein Phosphorylated stathmin promotes microtubule stability with an acetylated beta tubulin signature. Recruitment of the cellular phosphatase PP4c by MCPyV sT dephosphorylates stathmin and promotes microtubule catastrophe, resulting in a more motile cell phenotype associated with metastasis.

Merkel cell carcinoma (MCC) is a rare but highly metastatic neuroendocrine skin cancer [1]. Approximately 80% of MCC are caused by the recently described Merkel cell polyomavirus (MCPyV). Since its discovery in 2008 by the Chang and Moore laboratory, this small DNA virus has been the subject of intensive research. In particular, efforts have focussed on elucidating the mechanisms by which the virus encoded tumor antigens drive cancer progression. Whilst both the small T (sT) antigen and a truncated form of the Large T (LT) antigen are required for MCC cell survival and proliferation [2], expression of sT-antigen is sufficient to transform rodent fibroblast cells [3]. The recent generation of a transgenic mouse expressing sT-antigen under the control of an epidermis specific (keratin-5) promoter confirmed the oncogenic potential of this protein *in vivo* and should now provide a powerful tool to confirm cell based studies and aid in the identification of the mechanisms of transformation [4]. Undoubtedly, these studies have significantly increased our understanding of the molecular basis by which MCPyV drives MCC development, however, there has remained a frustrating lack of information regarding the highly metastatic nature of MCC. In a recent publication, we have begun to address this deficit and highlight a link between sT-antigen expression and cell motility [5]. Using a quantitative proteomic approach, we found levels of microtubule regulatory proteins enriched in a stable cell line inducibly expressing the sT-antigen. Microtubules are essential components of the cytoskeleton and play a crucial role in a number of cellular functions ranging from mitosis to cell polarity. Both the assembly and dynamics of microtubules are exquisitely controlled and are often deregulated in cancers. One of the most prominent microtubule regulators is stathmin, also known as oncoprotein-18. Stathmin overexpression is a feature of multiple cancer types and correlates with high metastatic potential. Levels of stathmin were significantly higher in Merkel cells expressing sT-antigen and in MCC tumor biopsy samples. Given the critical role of stathmin in promoting microtubule destabilisation, which is known to promote a more motile phenotype in cells, we compared levels of cell motility and migration between control and sT-antigen expressing cells. Using an Incucyte kinetic live cell imaging system we showed that sT-antigen expression increased cell motility. Moreover, using scratch assays we could clearly demonstrate changes in the migratory behaviour of cells containing sT-antigen. This phenotype was likely due to the deregulation of stathmin function. Specifically, stathmin localisation was modified in sT-antigen expressing cells. Compared to the diffuse, cytoplasmic staining observed in control cells, stathmin was sequestered to a perinuclear localisation, which co-localised with a population of sT-antigen. Importantly, the halo-like redistribution was co-incident with a decrease in levels of acetylated β-tubulin, a marker of microtubule stability. Together these data are indicative of a destabilised microtubule network [5]. Using small molecule inhibitors that target virus proteins or the host factors they subvert is a promising avenue to disrupt virus-associated oncogenesis. Accordingly, we took advantage of clinically tested taxanes to confirm that microtubule destabilisation was necessary for sTantigen mediated cell motility. Addition of Paclitaxel strikingly slowed down cell mobilisation and migration of cells expressing sT-antigen, with minimal impact on control cells. Whilst the clinical efficacy of taxanes is often constrained by acquired resistance mutations, the possibility of developing alternative inhibitors of sTinduced migration might be a useful avenue to pursue. In experiments to understand the molecular mechanisms for microtubule destabilisation we demonstrated that levels of stathmin phosphorylation were significantly lower in cells expressing sT-antigen compared to controls. The reversible phosphorylation of stathmin controls its biological activity by reducing its affinity for tubulin and hence preventing microtubule disassembly. Given that the regulation of protein phosphatase activity is believed to be a principal mechanism by which polyomaviruses manipulate intracellular signalling pathways, we investigated whether such enzymes were implicated in the observed microtubule destabilisation and cell motility. In stark contrast to other polyomaviruses, which mediate a number of their functions via a conserved interaction with PP2A, MCPyV sT-antigen has been shown to bind to several phosphatase subunits, including PP4c [6]. Using a panel of sT-antigen mutants we clearly demonstrated that the interaction with PP2A is dispensable for microtubule destabilisation, and that binding to PP4c is necessary for the observed dephosphorylation of stathmin (Figure 1). Differences in phosphatase sub-unit binding may help to explain some of the unique functions of MCPyV sTantigen compared to other polyomaviruses and warrants a greater scrutiny of the binding partners of sT-antigens from other polyomaviruses. In this light, recent findings now suggest that PP4c binding is conserved with the sTantigen of Simian Virus 40 (SV40) [7] but not the related BK and JC viruses (our unpublished data).

In summary, our study highlights a potential mechanism by which tumor antigen expression may enhance the migratory and invasive phenotype of MCC cells and further emphasises the critical role of stathmin in metastasis. It further distinguishes the sT-antigen of MCPyV from other polyomaviruses through its utilisation of alternative phosphatase binding partners. Importantly, it may provide a target for novel antitumor therapies to treat MCC.
